# Long Covid: conceptualizing the challenges for public health

**DOI:** 10.1093/pubmed/fdac153

**Published:** 2023-05-02

**Authors:** Jai Prashar

**Affiliations:** University College London Medical School, 74 Huntley St, London, WC1E 6DE, UK

**Keywords:** communities, COVID-19, public health

## Abstract

**Background:**

Long Covid has caused significant disruption to public services, economies and population health worldwide, but no single public health approach has proven effective in its management. This essay was the winning entry for the Faculty of Public Health’s Sir John Brotherston Prize 2022.

**Methods:**

In this essay, I synthesize existing literature on public health policy in long Covid, and discuss the challenges and opportunities posed by long Covid for the public health profession. The utility of specialist clinics and community care, in the UK and internationally, is examined, as well as key outstanding issues relating to evidence generation, health inequality and defining long Covid. I then use this information to inform a simple conceptual model.

**Results:**

The generated conceptual model integrates community- and population-level interventions; key areas of identified policy need at both levels include ensuring equitable access to long Covid care, developing screening programmes for high-risk populations, co-production of research and clinical services with patients, and using interventions to generate evidence.

**Conclusions:**

Significant challenges remain in the management of long Covid from a public health policy perspective. Multidisciplinary community-level and population-level interventions should be employed with a view to achieving an equitable and scalable model of care.

In May 2020, some patients recovering from infection with COVID-19 began to describe complex, persistent symptoms. Issues ranging from intense fatigue and breathlessness to debilitating confusion and joint pain were reported, but recognition from the scientific and medical communities was not forthcoming.^1^ By August, anecdote had become tentative evidence,^2^^,^^3^ and ‘long Covid’ had started to emerge as a concerning entity (Fig. 1). Two years later, long Covid is firmly embedded in the international consciousness, with a recent meta-analysis^4^ of prevalence studies suggesting that the condition may have already affected over 100 million individuals worldwide. Protracted disability is common—with as many as 91.8% of long Covid sufferers experiencing at least 35 weeks of symptoms in one prospective, international cohort^5^ and permanent organ damage having been observed in young, low-risk individuals.^6^ However, understanding of the disease processes that underpin long Covid remains poor. Similarly, the search for an effective treatment has not yet yielded results, although multiple large-scale randomized controlled trials are currently underway.^7^

Long Covid has already placed a considerable burden on public services. In one large study of hospitalized individuals, published in the *BMJ*,^8^ nearly a third required re-admission due to post-Covid multi-organ damage—suggesting that long-term and acute care services may bear the brunt of long Covid-related illness for many more years to come. In combination with other effects, such as an increase in psychiatric morbidity^9^ and depletion of the workforce,^5^ there is a strong impetus for long Covid to be addressed at public health level. There has been significant investment in infrastructure for post-Covid care in the UK—with the NHS having recently committed £100 million to long Covid assessment, rehabilitation, data collection and education in 2021/2022.^10^ The UK’s public health response has largely been underpinned by the development of specialist long Covid ‘clinics’, with additional funding for guided self-management and primary care.^10^

**Fig. 1:**
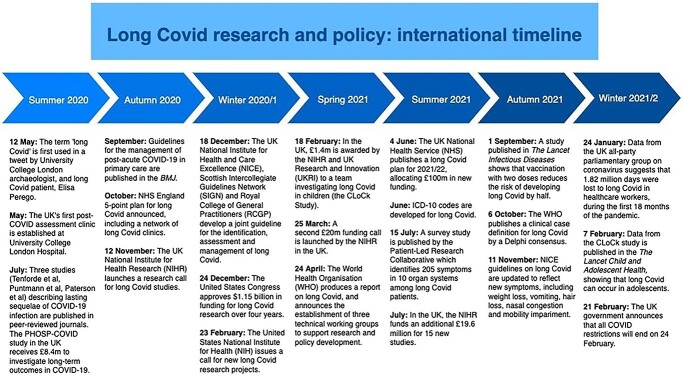
Long Covid research and policy timeline, Summer 2020—Winter 2021/2022.

Recent policy movement away from suppression of COVID-19 and towards an ‘endemic Covid’ approach is likely to result in a new surge of long Covid in the general population, amplifying the public health burden. Even mild and moderate COVID-19 infection have been shown to precipitate long Covid,^11^ which has concerning implications for populations. These challenges, like the acute pandemic, will span multiple countries, healthcare systems and communities—necessitating a highly adaptable and holistic approach to intervention. There are still no clear answers to the question of how public health should approach the issue of long Covid. Existing models and frameworks, whether of transmissible infectious disease,^12^ or chronic illness^13^ likely do not fully explain the complex determinants of outcomes in an epidemic, chronic disease like long Covid—nor do they recognize the need to develop active management strategies and interventions at local or national level. The lack of guiding principles or a clear evidence base has arguably given rise to an *ad hoc*, resource-intensive approach to care in the UK. With the majority of countries unable to invest millions in long Covid care, a pragmatic and sustainable approach to public health strategy is needed.

The aims of this work are 3-fold: first, to review practical examples and policy themes within the existing literature; second, to summarize challenges and learning points from the early public health response to long Covid; and third, to use this information to synthesize a basic conceptual model that can be used to guide public health approaches to long Covid.

An important caveat to this analysis is poor data availability. Although the scale and impact of long Covid are evident, longitudinal, population-level data collection and accurate determination of prevalence and healthcare utilization may be years away.^14^

Underreporting^15^ and heterogeneity in prevalence estimates across study populations^16^ pose challenges to surveillance at the most fundamental level. Equally, robust evaluations of acceptability and efficacy for interventions have not yet emerged. However, with long Covid becoming increasingly prevalent in the population, there is an imminent need to develop coherent, sustainable and effective public health strategies.

In line with methods described by Brady *et al.,*^17^ I conducted a literature review to identify concepts relevant to, and examples of, public health or policy responses to long Covid. Original research articles, reviews, editorials and commentaries in English were included. MEDLINE and EMBASE were searched to 10 February 2022 for the terms (*‘long Covid’ OR* (‘long-haul* AND Covid) *OR ‘post-Covid’ OR ‘ post-acute sequelae of SARS-CoV-2’ OR ‘PASC’*) *AND* (*‘policy’ or ‘public health’ or ‘population health’*)*.* Six hundred sixty-four citations were yielded.

Duplicates were removed, abstracts and then full texts were screened for relevance, and reference lists were manually searched to identify additional articles for inclusion. Thirteen articles were identified and coded thematically—these are reported in Table 1.

**Table 1:** Articles included in the synthesis

	Authors	Year	Title	Country^a^	Type	Theme(s)
1	Southwick *et al.*	2021	The role of digital health technologies in COVID-19 surveillance and recovery: a specific case of long haulers.	United States	Original research	Digital health, advocacy
2	Pye *et al.*	2021	A public health approach to estimating the need for long COVID services.	United Kingdom	Original research	Surveillance, care pathways, HNA
3	Fowler Davis *et al.*	2021	Assessing the acceptability of a co-produced long COVID Intervention in an underserved community in the UK	United Kingdom	Original research	Self-management, patient and public involvement
4	O’Brien *et al.*	2020	An integrated multidisciplinary model of COVID-19 recovery care.^18^	Ireland	Original research	Health system, care pathways
5	Ward *et al.*	2021	Global surveillance, research and collaboration needed to improve understanding and management of long COVID.	United Kingdom	Commentary	Inequality, risk factors, surveillance, research
6	Berger *et al.*	2021	Long COVID and health inequities: the role of primary care.	United States	Commentary	Economic burden, inequality, primary care, surveillance
7	Norton *et al.*	2021	Long COVID: tackling a multifaceted condition requires a multidisciplinary approach.^19^	United Kingdom	Commentary	Patient and public involvement, advocacy
8	Callard *et al.*	2020	How and why patients made Long Covid.	United Kingdom	Commentary	Patient and public involvement, advocacy
9	Brown *et al.*	2021	Conceptualizing long COVID as an episodic health condition.	United Kingdom	Commentary	Recognition, rehabilitation
10	Banerjee A.	2021	Long Covid: new wine in need of new bottles	United Kingdom	Commentary	Surveillance, care pathways, research
11	Glasper A.	2021	Strategies and policies to tackle the problems associated with long COVID.	United Kingdom	Editorial	Care pathways, surveillance
12	Wise J.	2021	Long Covid: WHO calls on countries to offer patients more rehabilitation.	United Kingdom	Editorial	Rehabilitation, surveillance, care pathways
13	Limb M.	2021	Covid-19: recognize long Covid as occupational disease and compensate frontline workers, say MPs.	United Kingdom	Editorial	Employment

^a^Country of origin of the majority of the authorship, or location in which the study was conducted.

No models or frameworks relating to long Covid and public health were found. Four original research articles, six commentaries and three editorials were identified. Ten articles originated from the United Kingdom, two from the United States and one from Ireland.

Conceptual models seek to diagrammatically represent proposed causal linkages among a set of concepts believed to be related to a particular problem or paradigm.^17^ The methods described by Earp and Ennett,^20^ Paradies and Stevens^21^ and Brady *et al.*^17^ were used to develop a basic conceptual model, describing considerations for public health policy and how they are interrelated (Fig. 2).

**Fig. 2:**
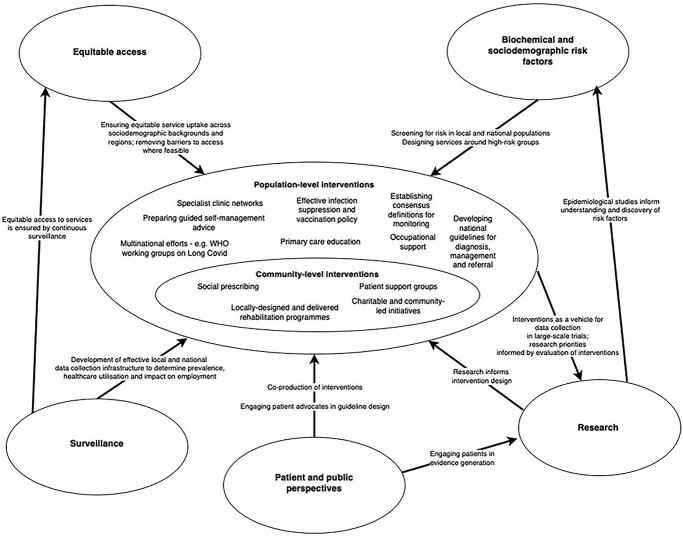
A broad conceptual model for public health policy in long Covid.

## Priorities, successes and challenges of existing approaches

Early public health approaches to long Covid in the UK have generally focused on action at the population level. The NHS Long Covid Plan for 2021/2022^10^ outlines an impressive set of proposals, including the expansion of clinics, primary care education, care co-ordination, the development of rehabilitation and self-management pathways and ensuring equitable access to care. However, the efficacy of these approaches remains unknown.

The data show that real-world utilization of specialist clinics is low. Fewer than 5500 referrals were made to long Covid clinics in England during the month beginning 22 November 2021,^22^ despite an estimated prevalence of 1.1 million long Covid cases^23^ over approximately the same period. Approximately 210 000 of these patients (Extrapolated from available data as functional impact was not reported by region. 244,000 people were in this category for the entire UK population, which was multiplied by 0.8589 to approximate the figure for England.) also believed that long Covid reduced their ability to undertake day-to-day activities ‘a lot’. Hence, 19% of patients likely experienced significant functional limitation, but only 0.48% of identified cases were ever referred to specialist clinics. These statistics may reflect more patients being successfully managed in primary care. However, with 99.5% of all cases—and at least 97% of all cases with considerable functional impairment—being managed outside of specialist clinics, there are likely to be barriers to accessing this care, ostensibly resulting in unmet need. The resulting, and possibly avoidable, impairment in functional ability may be reflected in the growing work absence rates attributed to long Covid.^24^

Berger *et al.*^25^ propose a ‘primary care-led’ response to long Covid, suggesting that holistic, biopsychosocial care is best delivered in general practice. Ward *et al.*^26^ support this approach, suggesting that although multidisciplinary services are able to manage patients more comprehensively, a move towards decentralized primary or community care may be more sustainable in the long term. It follows that such an approach is also likely to be more readily scalable to resource-limited care settings.

A primary care-based approach, however, is only likely to be of use insofar as primary care clinicians are equipped with the knowledge and understanding required to manage long Covid. Unifying national guidelines for long Covid are a cheap and effective intervention for ensuring recognition and management in primary care and have been developed for this purpose in multiple countries.^27–29^ However, low coding of long Covid in primary care in the UK—and large intra-practice variation—suggests that there may still be a poverty of understanding among many general practitioners.^15^ The rapid pace of change in the evidence base compounds the problem: as understanding grows, clinical education will need to be delivered continuously to reflect brisk progress in diagnosis and management.

Much of the global public health response, likely due to economic necessity, is limited to guideline provision and implementation of long Covid care into existing care pathways. Although specialist clinics exist in some European nations, the standard of care in these countries generally revolves around self-management and management in primary care.^28^ In India, tentative steps have been made towards guideline development,^29^whereas in China, a zero-Covid strategy appears to have overshadowed any consideration of long Covid care.^30^ It is perhaps too early to decide whether these approaches are capable of meeting unmet need.

Community-level intervention is rarely reported, and yet will likely be vital in meeting the specific needs of local populations. Fowler-Davis *et al.* report on the acceptability of a coproduced, virtual clinic for long Covid within a deprived community in the UK, finding that existing, nationally or regionally developed care pathways for long Covid may exclude some members of underserved populations.^31^ Rehabilitation, as described by Brown *et al.,*^32^ is a particularly important aspect of care for people living with long Covid, and is often delivered at community level. Interventions that reduce disability and enable patients to regain functional independence have important implications for service planning and wider population health,^33^ and therefore rehabilitation-based interventions—for example, physiotherapy, pulmonary rehabilitation and cognitive rehabilitation^34^—should be actively integrated into local care pathways.

Community-led charitable initiatives and social prescribing may offer another dimension to rehabilitation. The English National Opera (ENO) have designed ‘ENO Breathe’—a social prescribing programme using opera singing techniques to rehabilitate long Covid patients.^35^ In pilot testing, > 90% of participants perceived the programme to have had a positive impact on long-Covid-related breathlessness and anxiety—highlighting the value of such programmes in rehabilitative care.

National surveillance alone is unlikely to accurately characterize variable need between and within communities. Pye *et al.* describe a preliminary health needs assessment (HNA), whereby prevalence estimates for long Covid were applied to locally available data to inform the development of a post-COVID service.^36^ Considered needs assessments will be central to the development of acceptable, effective long Covid services and interventions that are well-suited to the communities they serve.

## Addressing biochemical and sociodemographic risk factors

Risk factors associated with the development of long Covid remain elusive, and yet understanding of these factors will likely underpin any preventative public health response. Data from an observational analysis by Sudre *et al.* suggest that the development of long Covid is associated with increasing age, body mass index, female sex and experiencing more than five symptoms in the initial week of illness.^37^ Interestingly, a preprint by Thompson *et al.* also suggests that ethnic minority origin is associated with significantly lower risk of developing long Covid,^38^ with an odds ratio (OR) of 0.32 [0.22-0.47]—despite the opposite being true of severe illness risk in acute COVID-19^39^ (which may in itself increase the risk of long Covid). The reasons for this apparent conflict, and the implications for ethnic minority populations, are as yet unclear.

Various studies have sought to characterize biochemical risk factors which might predict long Covid development at the early stages of illness. A January 2022 paper by Su *et al.* suggests that high serum levels of SARS-CoV-2 RNA or Epstein–Barr virus during acute infection, the presence of specific autoantibodies, or a pre-existing diagnosis of type 2 diabetes, were all positively associated with the development of long Covid.^40^ Similarly, Cervia *et al.* report the discovery of an antibody ‘signature’ which can be used to predict the development of long Covid with high confidence.^41^

So, what does a preventative intervention for long Covid look like? Determining who is likely to develop long Covid in advance may provide public health authorities with more accurate estimates of prevalence, enabling forecasting of healthcare utilization and informing equitable post-Covid service design. High-risk patients might also be directed to self-guided management information^42^ in advance, which may serve as an effective intervention to reduce the burden of mild cases presenting to general practice. Clearly, any effective measure preventing acute infection will also prevent the development of long Covid. Vaccination may also reduce long Covid risk after breakthrough infection. A case–control study by Antonelli *et al.* suggests that the odds of developing long Covid following infection are decreased by half in those who have received two doses of a vaccine (OR 0.51, *P* = 0.006).^43^ In health settings where complex care is economically or practically infeasible, seeking to increasing vaccination uptake (through methods well-described elsewhere^44^) is likely to be a powerful intervention.

## Recognizing and reducing inequality in long Covid care

Inequality is already a feature of long Covid care, with some communities experiencing significant barriers to access. Fowler-Davis *et al.*, through a Health & Wellbeing link worker embedded in a deprived community, identified long Covid patients in need of care but who had not presented to their GP.^31^ Barriers to presentation in primary care are common, widespread and may be associated with deprivation.^45^ This is of particular consequence when it is considered that primary care is generally the expected route of entry into long Covid care pathways. In underserved populations where primary care services are underutilized or inaccessible, consideration might be given to a social prescribing model—where underserved long Covid patients can be referred to link workers by a wide range of community teams; including social care workers, emergency services, allied health professionals and charitable organizations.

Ward *et al.* comment on how inequality is reflected in long Covid care internationally, with a specialized and multidisciplinary approach out of reach for many countries. As of January 2022, citizens of many countries do not have access to specialist facilities for assessment and treatment of long Covid at all.^28^ Multinational approaches may offer respite; the World Health Organisation (WHO) has set up technical working groups^46^ which aim to drive global understanding of long Covid and service development.

## Patient advocacy and involvement must be central to policy

Patients have been central to the development and recognition of long Covid. Callard *et al.* describe long Covid as a ‘patient-made illness’: patients coined the term, defined the disease and successfully advocated for its recognition.^1^ Advocacy groups continue to shape the long Covid policy debate, with organizations such as LongCovidSOS^47^ using patient voices to drive research and increase societal awareness. Patient-focused research can highlight important shared goals for service design: Ladds *et al.,* for example, in a qualitative study, identified key patient-focused quality principles for long Covid services.^48^ Some long Covid research has even been patient-led,^49^ representing a novel approach which should be integrated into research agendas where feasible.

Parallels have been made between long Covid and chronic fatigue syndrome/myalgic encephalomyelitis (CFS/ME), particularly in terms of the shared symptomatology, strong advocacy element and the uncertainty of the evidence base between these conditions.^50^ In CFS/ME, dissonance between patient advocacy groups and policy is a significant barrier to achieving consensus on treatment. Perhaps this highlights the practical importance of not just listening to, but actively incorporating the patient voice into the long Covid discourse—for example, by ensuring that advocacy and patient–public involvement (PPI) remain central to long Covid service and guideline design.

However, in lieu of an established evidence base, patient communities can be a source of misinformation—reinforcing the need to promote clear and unambiguous information about potentially harmful treatments. Southwick *et al.,* in an analysis of posts in the Reddit r/covidlonghaulers community, highlight the propagation of misinformation about long Covid treatment online.^51^ It should be considered whether the generation and uptake of misinformation are due to remediable factors, such as a lack of confidence in the rapidly changing scientific evidence base, or simply poor access to specialist care for long Covid.^52^ Integration of the patient voice into service planning may legitimize concerns and reduce the apathy that perpetuates the spread of misinformation.^53^

## Shaping research priorities

Research priorities in long Covid should be closely embedded into service design. Banerjee^54^ describes the benefits of an integrated approach to research and care, suggesting that long Covid services need to be supported by ongoing evidence generation. Increasing the evidence base for therapeutics in particular is recognized as a key priority for both patients and policymakers.^55^ Integrating trials into existing services^56^ may well achieve this, as well as helping to achieve cost-effectiveness by reducing unnecessary diagnostic testing.

A second key priority is designing a unifying definition for long Covid that may be used in research and monitoring. The WHO clinical case definition for long Covid was developed in October 2021 by way of a Delphi consensus,^57^ but is not entirely consistent with other definitions.^27^ Broad definitions can contribute to heterogeneity between prevalence estimates in research studies, leading to uncertainty about true prevalence figures. Effective surveillance—and subsequently, intervention—depends on being able to robustly recognize long Covid cases in the population.

With much of the world still in the throes of the pandemic, there has been a notable lack of long Covid research output from developing countries: in fact, a 2021 systematic review^58^ did not identify a single study from a low or medium income country (LMIC). With its extensive psychosocial and lifestyle impact, the lived experience of long Covid is inevitably different for patients in the UK or United States than for patients in Bangladesh or Nepal. Where feasible, local evidence generation—with input from local patient groups—can enable the development of locally applicable and culturally acceptable solutions.

## Developing a conceptual model

A broad conceptual model for public health policy in long Covid is presented in Fig. 2, incorporating the themes and challenges identified in the literature and discussed here. Determinants of care are linked to interventions at the centre of the model. Community interventions are placed within the wider context of public policy and population-level interventions, in line with the principles of the social ecological model described by Bronfenbrenner (1992).^59^ This recognizes the fact that interventions at the community level do not act in isolation, but are ultimately designed and experienced within the context of a rapidly evolving policy agenda at population level.

Aspects of the learning health system approach^60^ are also captured. For example, patients are engaged in evidence and policy generation, research informs intervention design, and interventions in turn are continuously evaluated to inform research priorities.

## Conclusion

As focus shifts to the ‘long tail’ of the COVID-19 pandemic, public health must act proactively to manage the risks posed by long Covid, both at community and population level. Vital challenges and lessons have been identified during the early response to long Covid in the UK and elsewhere, and these lessons should be heeded as public health strategies and approaches evolve with the generation of new evidence.

Close attention should be paid to the relative importance of general practice and community services. Initial approaches in many countries have favoured the adaptation of existing care structures over the development of a comprehensive specialist care network. It remains to be seen whether this is able to meet patient need; however, the apparent underutilization of specialist care in the UK is striking.

Reducing inequality and improving access to care should be a key priority, else long Covid care risks becoming a luxury for the privileged. Unequal access to care is likely to pose real threats to outcomes. Novel social prescribing approaches may help reduce barriers to access for underserved patients on a local level; equally, multinational approaches driven by organizations such as the WHO are likely to be powerful tools for raising awareness and driving service development globally.

Clinicians are currently required to make treatment decisions for long Covid patients that are based on poor quality evidence. Public health, with the input of patients and other stakeholders, will have a responsibility to shape and drive research agendas. Developing a robust definition for research and monitoring, enabling existing clinical infrastructure to generate new evidence and encouraging research in LMICs are all vital to forming a coherent picture of what long Covid is, and how it can be managed.

Finally, long Covid policy and care must be driven by the patient perspective. Involving patients from the conceptual stage of research, service and intervention design is likely to increase acceptability and impact. The influence of long Covid is significant, not just at the population or community level, but also on the individual. Sara Hawthorn,^61^ a woman with long Covid who was interviewed for the Guardian in 2021, describes the impact of her disease poignantly and powerfully: ‘*I was active, ran a business, danced a lot, walked, baked; all that is gone. Life is dull, small and boring. It’s hard to compare yourself with who you were before.*’ Long Covid is a disabling, multifaceted condition with implications at every level of society—and one which public health must be well-equipped to tackle in the months and years ahead.

## Notes

**Jai Prashar,** Final year medical student
